# Supplementary figures and images for: Antinociceptive Effects of Aaptamine, a Sponge Component, on Peripheral Neuropathy in Rats
Source: Mar Drugs. 2023 Feb 4;21(2):113. doi: 10.3390/md21020113 (PMC9963100; doi:10.3390/md21020113)

Fig. 8C

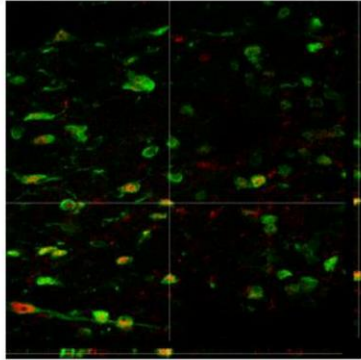

Fig. 8D

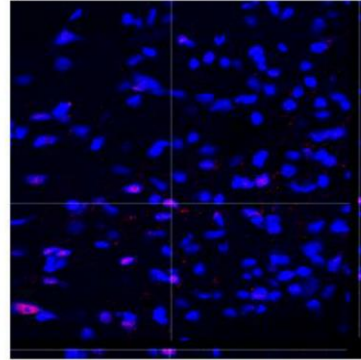

**Figure S1.** The uncropped images of Figure 8C,D.

Supplement: Supplementary file 1 [file marinedrugs-21-00113-s001.zip › marinedrugs-2184940-supplementary.pdf]
